# The longitudinal association between changes in lung function and changes in abdominal visceral obesity in Korean non-smokers

**DOI:** 10.1371/journal.pone.0193516

**Published:** 2018-02-23

**Authors:** Eun Kyung Choe, Hae Yeon Kang, Young Lee, Seung Ho Choi, Hee Joung Kim, Joo Sung Kim

**Affiliations:** 1 Department of Surgery, Healthcare Research Institute, Seoul National University Hospital Healthcare System Gangnam Center, Seoul, South Korea; 2 Department of Internal Medicine, Healthcare Research Institute, Seoul National University Hospital Healthcare System Gangnam Center, Seoul, South Korea; 3 Veterans Medical Research Institute, Veterans Health Service Medical Center, Seoul, South Korea; 4 Department of Internal Medicine, Konkuk University Medical Center, Seoul, South Korea; 5 Department of Internal Medicine, Liver Research Institute, Seoul National University College of Medicine, Seoul, South Korea; Federal University of Pelotas, BRAZIL

## Abstract

Obesity, particularly abdominal obesity, might be related to decreased lung function. We aimed to investigate whether obesity indices are associated with forced expiratory volume in one second (FEV1) and forced vital capacity (FVC) in asymptomatic non-smokers through a longitudinal cohort study. The clinical records of 1,145 subjects (428 males, mean age 52.3 years) who underwent a comprehensive health evaluation, including spirometry and abdominal fat computed tomography, at least twice between 2007 and 2014 were retrospectively reviewed and analysed. The mean follow-up period was 1,105 days (over 3.0 years). The baseline total adipose tissue (TAT) and visceral adipose tissue (VAT) were inversely associated with both FEV1 and FVC (*P* < 0.05). The longitudinal study found that increasing TAT and VAT were significantly related to decreasing FEV1 and FVC, whereas decreasing TAT and VAT were related to increasing FEV1 and FVC in both males and females (*P* < 0.05). The strength and consistency of these associations were clearer in males than in females. However, no significant relationship was found between changes in subcutaneous adipose tissue and changes in lung function. In Korean non-smokers, longitudinal changes in abdominal visceral fat were found to be inversely related to changes in lung function over a mean period of three years. These results suggest that decreasing abdominal visceral obesity could increase lung function despite ageing.

## Introduction

Obesity is associated with many health-related problems, including respiratory diseases [1,2]. Several studies have suggested that obesity, particularly abdominal obesity, is related to decreased pulmonary function [3–7]. However, previous studies have used body mass index (BMI), waist circumference (WC), and abdominal height as surrogate markers for abdominal adiposity because it is difficult to directly measure the abdominal fat distribution [4,5,8].

Fat tissue has been measured precisely through magnetic resonance imaging, dual-energy radiography absorptiometry, and computed tomography (CT). Abdominal adiposity consists of visceral adipose tissue (VAT) and subcutaneous adipose tissue (SAT). Compared with SAT, VAT is known to secrete cytokines and growth factors that stimulate the development of obesity-related pathological conditions [9,10]. The fat tissue compartment that is more strongly associated with pulmonary function remains unclear due to limited data. In a previous cross-sectional study in Korea, the amount of adipose tissue, TAT, SAT, or VAT, was inversely related to lung function among non-smokers [3]. In one study, visceral fat was negatively correlated with forced expiratory volume in one second (FEV1) and forced vital capacity (FVC) in non-diabetic men [6]. However, previous studies included only a small number of subjects or had cross-sectional designs. Consequently, the results are inconsistent regarding the association between abdominal obesity and pulmonary function [3,6,7].

Therefore, we aimed to investigate the relationship between abdominal obesity indices and pulmonary function with baseline data and to determine whether changes in abdominal obesity are associated with changes in pulmonary function in asymptomatic non-smokers through a longitudinal cohort study.

## Methods

### Study population

We performed a retrospective cohort study. The clinical records of 3,181 consecutive subjects who received a comprehensive health evaluation, including spirometry and abdominal fat CT, at least twice between January 2007 and December 2014 at the Seoul National University Hospital Healthcare System Gangnam Center were reviewed. The subjects who visited our centre were self-recruited through routine health check-ups. In most cases, the examinations and tests were performed on the same day. We excluded 2,036 subjects who were current or former smokers or had a history of lung cancer, tuberculosis, chronic obstructive pulmonary disease, or abnormal liver or kidney function. Finally, 1,145 subjects were enrolled in the study and subjected to further analysis. The study data included information provided by a questionnaire, anthropometric assessment results, laboratory data, and abdominal adipose tissue areas measured by CT. The study protocol was approved by the Institutional Review Board of Seoul National University Hospital (IRB No. H-1505-043-670). The board waived informed participant consent. This study was conducted in accordance with the Declaration of Helsinki.

### Measurements

The laboratory evaluation included total cholesterol (TC), triglycerides (TG), low-density lipoprotein (LDL) cholesterol, high-density lipoprotein (HDL) cholesterol, C-reactive protein (CRP), and fasting glucose and haemoglobin A1c (HbA1c) levels. Venous blood samples were collected before 10 AM after a 12-hour overnight fast. Height (cm) and body weight (kg) were measured using a digital scale. The BMI (kg/m^2^) was calculated as the weight divided by the height squared, and the WC was measured at the midpoint between the lower costal margin and the iliac crest by a well-trained nurse [11]. According to the International Obesity Task Force recommendation (WHO Western Pacific Region, 2000), the BMI categories were modified as follows: below-normal (< 23 kg/m^2^), overweight (between 23 and 25 kg/m^2^), and obese (> 25 kg/m^2^) [12,13]. Never-smokers were selected based on a self-administered medical questionnaire.

The subjects were examined in the supine position with a 16-detector row CT scanner (Somatom Sensation 16; Siemens Medical Solutions, Forchheim, Germany). The adipose tissue area was measured using a CT software programme (Rapidia 2.8; INFINITT, Seoul, Korea) that electronically determines the adipose tissue area in cm^2^ by setting the attenuation values for a region of interest within a range of -250 to -50 Hounsfield units, as previously described [14]. In summary, the TAT area was calculated using the region of interest drawn around the outer margin of the dermis. The VAT area was defined as intraabdominal fat bound to the parietal peritoneum or transverse fascia using a manual tracking method with cursors. The SAT area was defined as superficial fat in the abdominal and back muscles [14]. Spirometric tests were performed according to the American Thoracic Society recommendations by experienced technicians [15]. A dry rolling seal spirometer was used (Model 2130, Viasys Respiratory Care, Inc., San Diego, CA, USA). The FVC, FEV1, and FEV1/FVC were measured and are expressed as both absolute values (L) and predicted values (%) calculated from the formula based on the Korean population [3,16].

### Statistical analysis

The data are presented as the mean ± standard deviation in Table 1. We used linear regression and linear mixed models to predict the FVC and FEV1 from abdominal adiposity. Because no standard values exist to define a normal quantity of abdominal adipose tissue, we divided the baseline abdominal adiposity into four groups according to the tissue area (cut-off values in quartiles (cm^2^); male TAT 209.6, 264.5, 317.9; male SAT 105.9, 127.7, 160.8; male VAT 93.9, 129.9, 163.8; female TAT 198.3, 256.1, 321.5; female SAT 133.1, 171.8, 213.1; female VAT 53.9, 81.0, 112.5) to evaluate the relationship between abdominal obesity and pulmonary function, as shown in Table 2. All statistical analyses were performed separately according to gender because obesity values, pulmonary function values and other independent variables are significantly different between males and females. A multivariate analysis was used to control for the influence of age and other possible factors that may influence lung function.

**Table 1:** Baseline and follow-up characteristics of the subjects according to gender.

	Male (n = 428)		Female (n = 717)	
	Baseline	Follow-up	Baseline	Follow-up
Age (years, range)	52.1 ± 9.3 (30–81)	55.0 ± 9.3 (34–85)	52.4 ± 8.2 (25–75)	55.5 ± 8.3 (27–78)
Systolic BP (mmHg)	118.3 ± 12.7	118.7 ± 12.7	112.0 ± 14.6	113.6 ± 14.5
Diastolic BP (mmHg)	78.1 ± 10.2	77.9 ± 10.0	70.4 ± 10.7	71.7 ± 10.0
Height (cm)	170.4 ± 5.9	170.6 ± 5.9	158.4 ± 5.1	158.5 ± 5.1
Weight (kg)	70.7 ± 8.3	70.2 ± 8.4	56.0 ± 7.1	55.8 ± 7.2
Body mass index (kg/m^2^)	24.3 ± 2.4	24.1 ± 2.4	22.3 ± 2.7	22.2 ± 2.7
Waist circumference (cm)	87.0 ± 6.5	86.8 ± 6.7	80.9 ± 7.5	80.0 ± 7.8
Abdominal adiposity				
TAT (cm^2^)	266.4 ± 87.7	264.3 ± 87.3	261.7 ± 90.8	265.5 ± 93.3
VAT (cm^2^)	129.9 ± 52.9	127.3 ± 52.5	85.0 ± 40.4	86.2 ± 41.8
SAT (cm^2^)	136.6 ± 48.6	137.0 ± 49.1	176.7 ± 60.7	179.3 ± 63.0
VAT/SAT ratio	0.99 ± 0.40	0.97 ± 0.37	0.49 ± 0.20	0.49 ± 0.21
Total cholesterol (mg/dL)	191.4 ± 32.9	187.8 ± 34.3	198.5 ± 33.0	198.8 ± 34.0
Triglycerides (mg/dL)	110.5 ± 61.3	100.7 ± 55.8	84.9 ± 47.4	87.3 ± 48.4
HDL cholesterol (mg/dL)	51.4 ± 11.6	50.4 ± 11.0	59.5 ± 13.2	57.8 ± 12.7
LDL cholesterol (mg/dL)	123.0 ± 30.6	119.9 ± 29.9	123.3 ± 30.9	121.8 ± 30.5
Fasting glucose (mg/dL)	97.0 ± 15.5	97.9 ± 15.3	91.2 ± 15.1	94.1 ± 16.3
HbA1c (%)	5.8 ± 0.5	5.6 ± 0.6	5.8 ± 0.5	5.7 ± 0.5
CRP (mg/dL)	0.1 ± 0.2	0.1 ± 0.2	0.1 ± 0.6	0.1 ± 0.2
FVC (L)	4.23 ± 0.63	4.15 ± 0.65	3.03 ± 0.45	2.95 ± 0.46
FVC (% predicted)	96.6 ± 11.1	96.1 ± 11.2	99.0 ± 12.3	98.9 ± 12.5
FEV (L)	3.42 ± 0.55	3.32 ± 0.57	2.48 ± 0.40	2.38 ± 0.42
FEV1 (% predicted)	105.7 ± 13.1	105.3 ± 13.5	107.8 ± 14.2	107.2 ± 15.5
FEV1/FVC (%)	80.8 ± 6.1	80.0 ± 6.0	81.9 ± 6.1	80.7 ± 5.8

HDL, high-density lipoprotein; LDL, low-density lipoprotein; HbA1c, haemoglobin A1c; CRP, C-reactive protein; The data are presented as the mean ± standard deviation.

**Table 2:** Associations between baseline obesity indices and baseline lung function.

	FVC			FEV1			FEV1/FVC (%)		
Factor	β	SE	*P*-value	β	SE	*P*-value	β	SE	*P*-value
Male									
BMI (kg/m^2^)^1^	0.00552	0.01478	0.709	0.00430	0.01192	0.720	0.00369	0.15011	0.980
WC (cm)^2^	-0.01155	0.00887	0.194	-0.01044	0.00756	0.169	-0.04355	0.10895	0.690
TAT^3^	-0.13530	0.03884	< 0.001	-0.09543	0.03335	0.005	0.09882	0.48727	0.839
SAT^3^	-0.05224	0.03638	0.152	-0.02928	0.03109	0.347	0.15020	0.44858	0.738
VAT^3^	-0.13383	0.03299	< 0.001	-0.12032	0.02804	< 0.001	-0.37761	0.41621	0.365
Female									
BMI (kg/m^2^)^1^	0.01841	0.00724	0.011	0.00975	0.00599	0.104	-0.18724	0.10156	0.066
WC (cm)^2^	-0.00559	0.00422	0.186	-0.00407	0.00360	0.259	0.02355	0.06748	0.727
TAT^3^	-0.05271	0.02259	0.020	-0.04613	0.01928	0.017	-0.14070	0.36331	0.699
SAT^3^	-0.01011	0.01979	0.610	-0.00495	0.01691	0.770	0.20580	0.31670	0.516
VAT^3^	-0.06542	0.02111	0.002	-0.04588	0.01808	0.011	0.14834	0.34090	0.663

^*1*^ Adjusted for age, systolic blood pressure, glucose, TG, HDL-cholesterol, and C-reactive protein.

^*2*^ Adjusted for age, height, weight, systolic blood pressure, glucose, TG, HDL-cholesterol, and C-reactive protein.

^*3*^ Adjusted for age, height, weight, waist circumference, systolic blood pressure, glucose, TG, HDL-cholesterol, and C-reactive protein.

β, regression coefficient for each predictor; SE, standard error of β.

BMI, body mass index; WC, waist circumference; TAT, total adipose tissue; VAT, visceral adipose tissue; SAT, subcutaneous adipose tissue.

TAT, SAT, and VAT were divided into quartiles according to the tissue area.

Cut-off values of quartiles (cm^2^); male TAT, 209.6, 264.5, 317.9; male SAT, 105.9, 127.7, 160.8; male VAT, 93.9, 129.9, 163.8; female TAT, 198.3, 256.1, 321.5; female SAT, 133.1, 171.8, 213.1; female VAT, 53.9, 81.0, 112.5.

**Fig 1:**
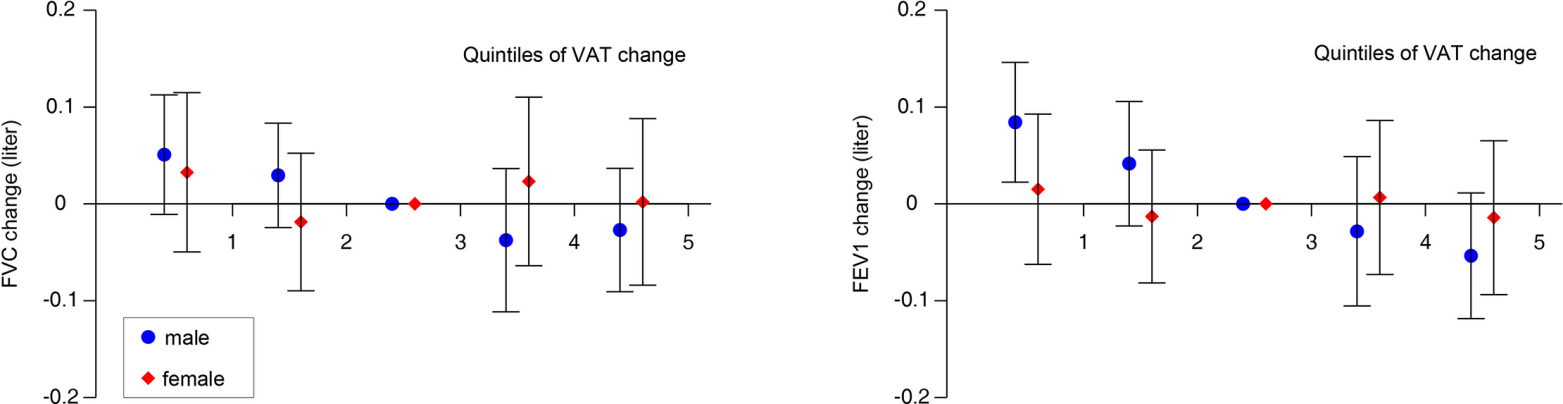
Changes in lung function according to changes in visceral adiposity. Longitudinal changes in FEV1 and FVC in quintiles of VAT change in males (circles) and females (diamonds). The middle quintile was used as a reference. Quintiles 1–2 include subjects with VAT loss, whereas quintiles 4–5 include individuals with VAT gain. The analysis was adjusted for baseline age, height, weight, WC, systolic BP, and fasting glucose, TG, HDL cholesterol, CRP level, and the follow-up period. Cut-off values in the quintiles of VAT change (cm2): males, -24.0, -6.6, 4.7, 17.2; females, -14.1, -3.2, 4.8, 15.0.

The longitudinal association between abdominal obesity and lung function was evaluated with the use of linear mixed models, as shown in Table 3. Model 1 was adjusted for baseline age, height, weight, waist circumference, systolic blood pressure, glucose, TG, HDL-cholesterol, C-reactive protein, and follow-up period. For model 2, significant variables were selected from model 1 using the likelihood ratio test based on the maximum likelihood method. In Fig 1, VAT changes were stratified into five groups according to their longitudinal changes (cut-off values in quintiles of VAT change (cm^2^): males, -24.0, -6.6, 4.7, 17.2; females, -14.1, -3.2, 4.8, 15.0) to determine whether changes in abdominal fat were associated with changes in pulmonary function. Statistical analyses were performed using the Statistical Package for Social Sciences, version 22.0 (SPSS, Inc., Chicago, IL, USA), and R statistical software, version 3.2.2. (R development Core Team; R Foundation for Statistical Computing, Vienna, Austria). Statistical significance was established for two-sided *P*-values < 0.05.

**Table 3:** Longitudinal associations between abdominal obesity and lung function using a linear mixed model.

	Model 1			Model 2		
Factor	β	SE	*P*-value	β	SE	*P*-value
FVC (L)						
Male						
TAT (cm^2^)	-0.00115	0.00035	< 0.001	-0.00140	0.00032	< 0.001
SAT (cm^2^)	-0.00089	0.00057	0.116	-	-	-
VAT (cm^2^)	-0.00153	0.00047	0.001	-0.00116	0.00036	0.002
Female						
TAT (cm^2^)	-0.00038	0.00019	0.047	-	-	-
SAT (cm^2^)	-0.00013	0.00023	0.579	-	-	-
VAT (cm^2^)	-0.00115	0.00038	0.002	-0.00144	0.00036	< 0.001
FEV1 (L)						
Male						
TAT (cm^2^)	-0.00105	0.00029	< 0.001	-0.00131	0.00027	< 0.001
SAT (cm^2^)	-0.00079	0.00048	0.101	-	-	-
VAT (cm^2^)	-0.00144	0.00040	< 0.001	-0.00176	0.00038	< 0.001
Female						
TAT (cm^2^)	-0.00028	0.00016	0.082	-0.00031	0.00015	0.044
SAT (cm^2^)	-0.00019	0.00020	0.333	-	-	-
VAT (cm^2^)	-0.00061	0.00032	0.057	-0.00068	0.00031	0.030

Model 1 was adjusted for baseline age, height, weight, waist circumference, systolic blood pressure, glucose, TG, HDL-cholesterol, C-reactive protein, and follow-up period.

Model 2 was adjusted for variables that were selected using the likelihood ratio test based on the maximum likelihood method.

β, regression coefficient for each predictor; SE, standard error of β.

TAT, total adipose tissue; VAT, visceral adipose tissue; SAT, subcutaneous adipose tissue.

## Results

### Baseline and follow-up characteristics of the study population

The baseline and follow-up characteristics of the study population are shown in Table 1. A total of 1,145 subjects (428 males, baseline mean age 52.3 ± 8.6 years) were analysed in this study. The mean follow-up period was 1,105 days (over 3.0 years, 217–2,199 days). The baseline median BMI and WC values among males and females were 24.2 kg/m^2^ and 87.0 cm and 22.2 kg/m^2^ and 80.9 cm, respectively. The proportions of male subjects characterised as below-normal, overweight, and obese were 28.6%, 34.4%, and 37.0%, respectively. Additionally, the proportions of female subjects defined as below-normal, overweight, and obese were 63.1%, 22.6%, and 14.3%, respectively. During the follow-up period, the BMI and WC decreased in 53.0% and 54.1% of the subjects, respectively, compared with the baseline values. Although the TAT of the abdomen in males (266.4 cm^2^) was similar to that in females (261.7 cm^2^), the VAT/SAT ratio differed: specifically, the ratios were 0.99 in males and 0.49 in females. The baseline FVC and FEV1 values were 4.23 ± 0.63 L and 3.42 ± 0.55 L in males and 3.03 ± 0.45 L and 2.48 ± 0.40 L in females, respectively. The baseline FEV1/FVC in males and females was 80.8 ± 6.1% and 81.9 ± 6.1%, respectively.

### Association between baseline obesity indices and baseline lung function

The relationships between baseline obesity indices and baseline lung function are displayed in Table 2. In males and females, the baseline TAT and VAT values were inversely associated with both the FEV1 and FVC. However, baseline SAT was not related to lung function. In females, the BMI was directly associated with the FVC. The baseline obesity indices were not associated with the FEV1/FVC ratio.

### Association between changes in abdominal obesity and changes in lung function

A study to investigate the association between changes in abdominal obesity and changes in lung function was performed (Table 3). With an approximately three-year follow-up, changes in lung function were inversely related to changes in adiposity. In model 1, after adjusting for the follow-up period, baseline age, height, weight, WC, systolic blood pressure (BP), and fasting glucose, TG, HDL cholesterol, and CRP levels, an association was found between changes in visceral adiposity and changes in lung function in the multivariate analysis. In models 1 and 2, longitudinal changes in TAT and VAT were inversely associated with changes in FEV1 and FVC in both males and females. However, SAT was not associated with FVC or FEV1 in either model 1 or 2.

Fig 1 illustrates the relationship between changes in visceral adiposity and changes in lung function. VAT changes were stratified into five groups according to their longitudinal changes to determine whether changes in abdominal fat were associated with changes in pulmonary function. In subjects with increased VAT, lung function was deteriorated, whereas lung function was improved in those with reduced VAT. This pattern was observed in both genders, but the strength and consistency of these associations were more obvious in males than in females.

## Discussion

In this study, lung function was significantly associated with abdominal obesity, particularly visceral fat, in asymptomatic non-smokers after adjusting for variables. Over a three-year period, increased visceral adiposity was associated with decreased lung function, whereas decreased visceral adiposity was associated with improved lung function in both males and females. There were no statistically significant relationships between changes in SAT and changes in lung function. In this retrospective cohort study, the mean follow-up results, such as the mean BMI, WC, and cholesterol, triglyceride, and HbA1c levels, were improved compared with the baseline metabolic values, particularly in males. This phenomenon was due to annual health check-ups and management despite ageing, but it is unclear why this trend was not observed in females, although one possible explanation could be the complex effects of menopause [17].

Several mechanisms have been suggested for how obesity affects lung function. Respiratory function is influenced by the interaction between the lungs, chest wall, and respiratory muscles. Abdominal obesity is known to reduce chest wall compliance, respiratory muscle function, and peripheral airway size [5,18–20]. Obesity is also known to have a greater effect on the FVC, which represents the lung volume, and a lesser effect on the FEV1, which represents expiration flow rates. A previous study showed that higher WC and BMI values are associated with significantly lower FVC values, but these values are only slightly associated with FEV1 values [5]. The mechanical effects of the intraabdominal pressure on the diaphragm have been suggested as an important reason for the relationship between central obesity and lung function. However, our data showed no significant association between the baseline BMI or WC and lung function, except the BMI in females. In the present study, a lower baseline BMI was associated with a significantly lower FVC in females. This finding might be due to the larger proportion of thin females in our study, and poorer lung function in thin body types might be due to a lower muscle mass [21]. In fact, this result confirms the limitations of the BMI and WC as measures of obesity. The BMI does not distinguish between muscle and fat and inaccurately predicts the percentage of body fat [22]. In addition, WC is predominantly an index of subcutaneous abdominal fat and not visceral fat [23]. Higher baseline TAT and VAT values, but not SAT values, were found to be associated with significantly lower FVC and FEV1 values in our study, which is consistent with previously reported data [6]. Therefore, our data showed that central obesity, particularly visceral obesity, has a greater effect on lung function than general obesity. This result was confirmed through a longitudinal study, which showed that changes in lung function were inversely associated with changes in TAT and VAT.

Another possible explanation for the relationship between lung function and obesity is the systemic inflammation triggered by adipose tissue. VAT is well known to be more metabolically active than SAT. VAT secretes or synthesises inflammatory cytokines, such as tumour necrosis factor-α and interleukin (IL)-6 [9,24,25]. In some studies, VAT and IL-6 were found to be related to CRP levels [26–28]. Elevated CRP or IL-6 levels are known to be associated with chronic inflammatory airway disease [3,29,30]. Furthermore, activated macrophages in adipose tissue are known to cause low-grade chronic inflammation [31–33]. A previous observational study showed that systemic inflammation was related to reduced lung function in non-smoking males [34]. Considering these data, our results showing the relationship between visceral adiposity and lung function could be accepted in terms of systemic inflammation.

A previous study showed that decreased adiposity is associated with increased lung function to a greater degree in males than females [35]. This pattern was observed in the present study, and the strength and consistency of these associations were more obvious in males than in females. These results can be explained by mechanical aspects: males have more abdominal fat than females, with a similar degree of obesity [35–37]. A greater deposit of fat in the abdominal region generates greater resistance to diaphragmatic contraction, disturbing ventilation mechanics [38]. In addition, several other possible explanations have been suggested, including differences in sex hormone levels, thresholds for the harmful effects of pulmonary irritants, or differences in the airway calibre between males and females [39,40]. In the present study, lung function was found to be influenced by VAT, and the gap between males and females was presumed to be due to the high VAT/SAT ratio in males.

The present study has several strengths. First, this was a longitudinal cohort study, unlike most of the previous cross-sectional studies of lung function and obesity. Second, this study included a relatively large number of participants in whom abdominal adipose tissue was directly measured with CT scans, unlike previous studies that used the BMI or WC as surrogate markers for abdominal adiposity. Third, our subjects were relatively healthy homogeneous participants. In addition, we included only non-smokers to eliminate any influences of previous or current smoking.

However, our study has several limitations. First, the subjects of this study were self-recruited for routine health check-ups, and this study was conducted in one centre. Therefore, our findings might not represent the general population. Second, although significant associations were found, the approximately three-year follow-up period was not sufficiently long to identify the long-term relationships between changes in obesity indices and lung function. Whether these changes are maintained long-term requires further investigation. Third, this study included relatively few obese people, particularly obese females. Thus, the relationship between lung function and abdominal obesity might be different for very obese persons. The implication of this study is that even in those who are not significantly overweight, decreasing abdominal fat may be effective in increasing pulmonary function. Further research is required for people who are very obese.

## Conclusions

In conclusion, increased abdominal visceral obesity is associated with decreased lung function (FVC and FEV1), whereas decreased abdominal visceral obesity is related to increased lung function in Korean non-smokers. This could be important information for planning lung function screenings and health check-up follow-ups for subjects with abdominal obesity. In addition, abdominal visceral obesity must be prevented and improved in asymptomatic subjects to also improve pulmonary function and metabolic disease. However, a long-term prospective cohort study simultaneously measuring cytokines and proinflammatory mediators produced by adipocytes might be helpful for identifying certain causal or temporal relationships between lung function and abdominal obesity.

## Abbreviations

BP: blood pressure
BMI: body mass index
WC: waist circumference
CT: computed tomography
TC: total cholesterol
TG: triglycerides
LDL: low-density lipoprotein
HDL: high-density lipoprotein
CRP: C-reactive protein
HbA1c: haemoglobin A1c
IL: interleukin
FEV1: forced expiratory volume in one second
FVC: forced vital capacity
TAT: total adipose tissue
VAT: visceral adipose tissue
SAT: subcutaneous adipose tissue
